# Collective choice fosters sustainable resource management in the presence of asymmetric opportunities

**DOI:** 10.1038/s41598-020-67757-1

**Published:** 2020-07-01

**Authors:** Laila Nockur, Laetitia Arndt, Johannes Keller, Stefan Pfattheicher

**Affiliations:** 1 Department of Social Psychology, Ulm University, Albert-Einstein-Allee 47, 89069 Ulm, Germany; 2 Department of Psychology and Behavioural Sciences, Aarhus University, Bartholins Allé 11, 8000 Aarhus C, Denmark

**Keywords:** Environmental economics, Psychology and behaviour, Sustainability

## Abstract

Asymmetric distribution of opportunities among actors can reinforce the conflict between individual and collective interests in social dilemma situations. The present study investigates the interplay of asymmetric distribution of opportunities to consume resources and three choice systems: individual choice, median choice, and majority voting. Participants (*N* = 248) took part in a common resource game in groups of four under each of the three choice systems. We examined the average percentage taken of the resource as well as satisfaction and fairness ratings depending on the choice system in interaction with (a) whether the distribution of opportunities among group members was symmetric versus asymmetric, and (b) the status of an actor (advantaged versus disadvantaged) within asymmetric groups. Both implemented collective choice systems (median choice and majority voting) increased sustainable resource management, especially in asymmetric groups, by restricting overconsumption of advantaged individuals, as well as satisfaction and fairness ratings. Collective choice increased collective welfare by increasing profits of disadvantaged individuals and members of symmetric groups. The results indicate that in the presence of asymmetric distribution of opportunities, collective choice is a means to reconcile the conflict between individual and collective interests in social dilemmas and to foster sustainable resource management.

## Introduction

The consumption of limited, shared resources represents a social dilemma, a situation where individual and collective interests conflict. Often the groups of actors, be that of individuals or collectives like states, are characterised by asymmetric distribution of opportunities (e.g., financial), such that some actors have privileged access to common resources while others are disadvantaged^1–3^, which can make such dilemma situations even more intricate. Prior work revealed that asymmetric distribution of opportunities within groups is associated with lower levels of cooperation^4–10^ and increases the risk of failing to reach a collective goal, such as jointly raising a target sum of money to avoid losing the money altogether^6,7,11^.

As asymmetry between actors can be a challenge for cooperation, a serious question emerges about how to establish cooperation in the presence of asymmetric distribution of opportunities. We propose implementing a decision process at the collective level to foster sustainable resource management in the presence of asymmetric opportunities. Specifically, the current investigation examines the effectiveness of two collective choice systems in fostering sustainable management of common resources in a common resource game, when involved actors’ opportunities to harvest from the resource are distributed symmetrically versus asymmetrically. Additionally, we examine how satisfied the actors are with each choice system in interaction with (a) whether actors within groups have symmetric versus asymmetric opportunities to harvest from the resource, and (b) the status of an actor (advantaged or disadvantaged) within asymmetric groups.

The consumption of shared resources constitutes a social dilemma, because sustaining the resource (so that many can profit from it) requires individual restraint^12,13^. Although groups of cooperative individuals, who restrict their consumption behaviour to benefit the collective, profit more than groups of defectors, individuals can earn most if they do not cooperate but instead exploit the common resource^14^. Usually, the consumption of resources is determined by individual choice, that is, individuals decide simultaneously and in isolation. However, individual choice fosters overexploitation of common resources^14,15^ and failure to reach a collective goal^6,7,11^. Evidently, the question is whether collective (rather than individual) choice may facilitate pursuing interests of the collective by forcing individuals to reach a decision at the group level. Indeed, voting on an extraction level has been documented as one feasible way to increase sustainable resource use^14,16,17^.

So far, most studies on social dilemma situations involved groups of individuals with symmetric opportunities. However, actors in the real world in many cases do not have the same preconditions^18–20^ with considerable consequences for cooperation. An asymmetric distribution of opportunities in social dilemma situations can induce ambiguity on how to act—should all group members behave in the same way or should the asymmetry be reflected in their behaviour? Although individuals seem to strive for equal costs and payoffs^21–23^, asymmetry induces uncertainty about the fair solution^24–27^. In fact, asymmetric opportunities promote egocentric assessments of fairness^28^. Overall, cooperation levels are typically lower in groups characterised by asymmetric distribution of opportunities among actors^4–10,29^. Voting on a binding common extraction level could overcome the negative effects of asymmetry as outlined in what follows.

Voting is a commonly used mechanism to reach a collective decision as a function of individual interests. Empirical evidence suggests that voting on a common contribution or extraction level can increase cooperation and the sustainable use of common resources^14,16,17,21,30,31^. The specific voting system influences the likelihood of reaching a decision and its outcomes. For example, agreement is more often reached when determined by a majority rule compared to an unanimity rule^17,31^. In general, cooperation depends on whether the actors reach an agreement^21,31^ and whether the voting is binding or transgressions can be punished^32^.

In the present study, we examine two specific forms of collective choice, the implementation of a median choice system^14,30^ and the implementation of a majority voting system^32,33^. In the implemented median choice system, each group member suggests an extraction level, and the median of these suggestions is implemented for all group members. The system leads to sustainable resource use when there is a majority of cooperators who can overrule a minority of non-cooperators^14^. In the implemented majority voting system, each group member proposes an individual extraction level for each group member, including themselves. All group members then vote on these proposals. If a majority votes for the same proposal, it is implemented. If no proposal reaches a majority, each group member can decide individually how much to extract in that period.

Extraction decisions should take into account two factors: (a) development of the resource, and (b) distribution of extraction levels across group members. Long-term interest should always be to sustain the resource preserving enduring extraction possibilities. Therefore, the group should not exceed the sustainable rate of consumption that allows the resource to regenerate. The question remains about how extractions from the resource should be distributed among group members. The fairness principle of equality suggests equal rates for every group member; however, it is the interest of every group member to maximise his or her own share. Therefore, individuals could win the most if they voted on a solution that granted them the maximum while excluding other group members from consumption (e.g., by sharing the total extraction between a subgroup of group members). The majority voting system implemented in the present study in principle allows for differences in group members’ extraction levels, but as individuals usually strive for equal payoffs^21^, suggesting equal extraction levels is probably more likely to reach a majority in contrast to suggestions that favour individual group members. Independent of the extraction level, the implemented median voting system guarantees equal payoffs for all group members, rendering irrelevant the factor of distribution among group members. When there is a majority of cooperators, both systems should lead to more sustainable resource management compared to individual choice. However, asymmetric opportunities could make it more difficult to reach a common decision in the majority voting system, so the median choice system could be most efficient in promoting sustainable resource use. Regarding the evaluation, on the other hand, the majority voting system with the two-step process of proposing extraction levels for every group member and then voting on these proposals offers more participation in the decision process and might therefore be perceived more positively than the median choice system.

The present investigation advances the understanding of behaviour in social dilemmas by highlighting the importance of considering asymmetric opportunities of actors in interaction with situational constraints. In the real world, there are huge differences in (e.g., financial) opportunities between individuals or other parties like states or communities that influence resource consumption^1–3,18–20^. We examine how asymmetric opportunities interact with collective choice systems regarding sustainable behaviour in a common resource game. Asymmetry likely influences the effectiveness of structural solutions to dilemmas (i.e., voting systems): Symmetric actors profit equally from a collective decision; in asymmetric groups, however, different group members might be advantaged by different choice systems, which could make it more difficult to reach a common decision in asymmetric groups. On the other hand, we propose that in the presence of asymmetric opportunities in particular, collective choice can be a means of improving resource management by preventing over-consumption by advantaged group members. We examine sustainable resource management within three specific choice systems—individual choice, median choice, and majority voting—across groups with symmetric versus asymmetric opportunities. We argue that the median choice system is effective if there are enough cooperative individuals within the group. However, the median choice system is restrictive simply by excluding the extremes, and it does not allow for any variations among individuals’ profit. The majority voting system, on the other hand, seems to be a more realistic alternative, allowing more participation in the decision process and variation in individuals’ profit. However, whereas a common decision in the median choice system is guaranteed, the majority voting system leaves room for failure to reach a common decision. Therefore, we seek to answer the question regarding which of the implemented collective choice system is best suited to foster sustainable resource management in the presence of asymmetric opportunities.

From an applied perspective, we want to emphasise that it is not sufficient to demonstrate that a system is effective in promoting sustainable resource management for it to be implemented; it is also necessary that the actors involved support the system. We therefore include in our analyses an evaluation of each system regarding satisfaction with the outcome and fairness perceptions as a second dependent variable. It remains an open question how collective choice systems that promote a common decision on how to behave in a dilemma are being evaluated and how these evaluations are influenced by symmetric versus asymmetric opportunities within the group, on one hand, and by the status of the actor (advantaged versus disadvantaged) on the other.

To examine the interplay of asymmetric opportunities within groups and different choice systems on sustainable resource management, participants took part in a common resource game in groups of four (see “Methods” section). Participants played six periods of each choice system, namely individual choice, median choice, and majority voting (within-subjects factor). Between subjects, we varied whether group members had symmetric or asymmetric opportunities to harvest from the common resource. Whereas individuals in symmetric groups could extract up to one fourth of the resource available, in asymmetric groups, two advantaged group members could extract more units in each period (up to one third of the resource available) than two disadvantaged group members (up to one sixth). Based on prior evidence, we derived the following hypotheses:

- Asymmetry within groups fosters unsustainable resource use.
- Both collective choice systems, median choice and majority voting, foster more sustainable resource use compared to individual choice.

We further propose that the collective choice systems can overcome negative effects of asymmetry and therefore can prove especially effective in asymmetric groups. Both collective choice systems diminish the influence of the extreme—in the median choice system the two extreme proposals are discarded, and in the majority voting system proposals that favour individual group members are not likely to receive a majority. Therefore, both collective choice systems should be especially effective in altering behaviour of advantaged group members within asymmetric groups, for which more extreme extraction behaviour should be observed under individual choice. Regarding the interaction of asymmetry and choice system, we therefore expected the following:

(c) The collective choice systems have a greater effect in fostering sustainable resource use in asymmetric compared to symmetric groups.
(d) Collective choice should in particular restrict the resource consumption of advantaged group members compared to disadvantaged group members.

Additionally, we explored how each choice system is being evaluated by assessing ratings on outcome satisfaction and fairness perception after each choice system. One could expect differential effects of asymmetric opportunities on satisfaction and fairness ratings depending on choice system. As asymmetry fosters egocentric assessment of fairness^28^, advantaged group members might evaluate the individual choice system more favourably, while disadvantaged group members might prefer the collective choice systems. On the other hand, individuals seem to strive for equal costs and payoffs^21–23^, so all group members could evaluate the collective choice systems more favourably than individual choice if these systems promote more equal profits. Overall, we expected higher satisfaction and fairness ratings in the collective choice systems compared to individual choice, as these systems foster sustainable resource management and therefore higher profits in the long run.

## Results

### Extraction behaviour

**Figure 1:**
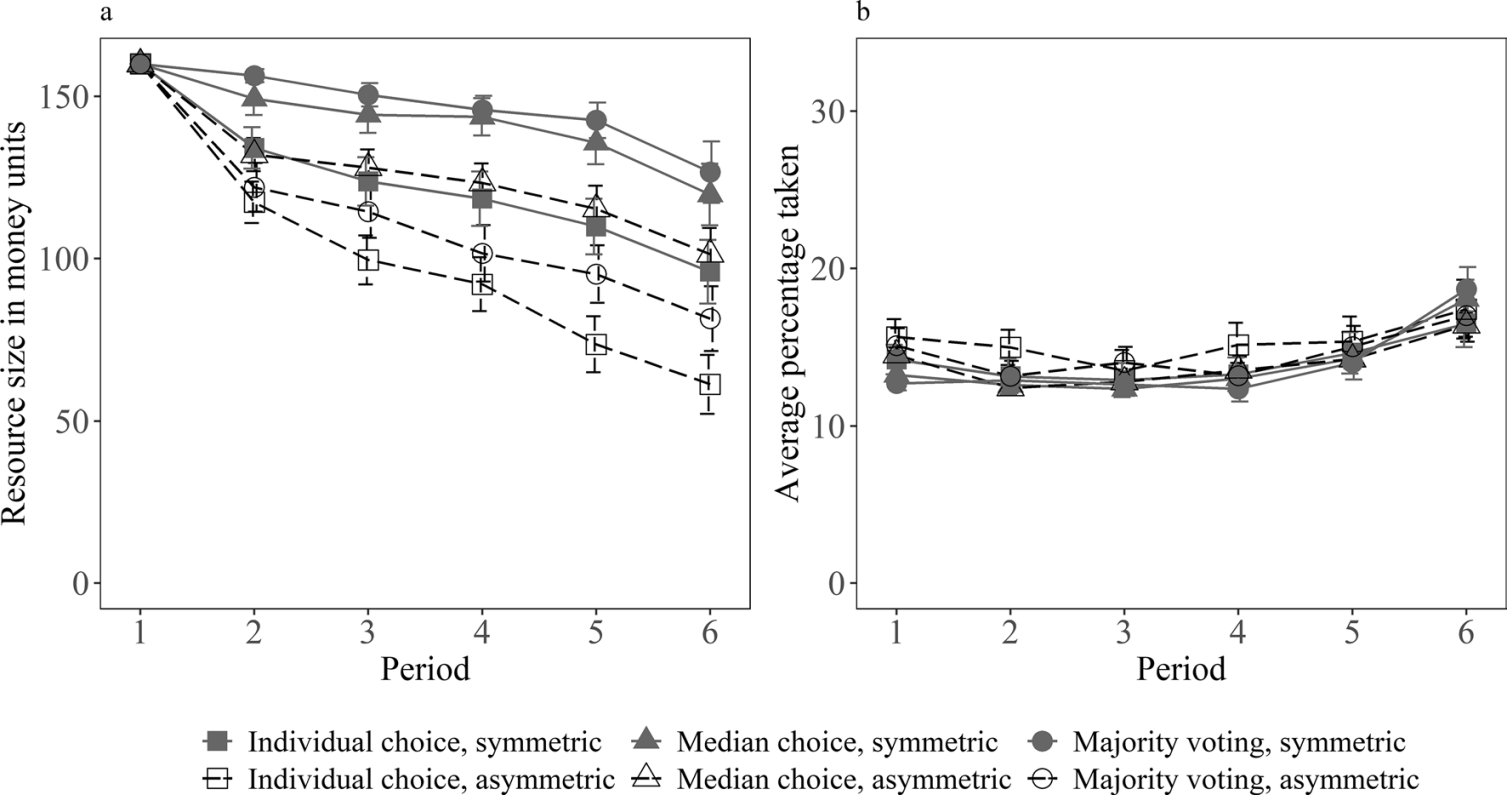
Resource size (**a**) and average percentage taken (**b**) depending on period, choice system, and asymmetry; error bars represent 95% confidence intervals.

As the resource doubled in size between periods, all group members could extract a rate of 12.5% in each period while sustaining the resource (see Methods section for more details). In all three choice systems, the average percentage taken significantly exceeded this threshold, which led to an overall decrease of the resource over the six periods (see Fig. 1a). Extraction rates were higher in early and late periods (see Fig. 1b and Supplementary Information for analyses on time trends in extraction over the six periods). Participants knew that they would play six periods under each choice system. Only few groups exhausted the resource in earlier periods (two under individual choice, one under median choice, five under majority voting). Many groups exhausted the resource completely in period six (five under individual choice, twelve under median choice, and 15 under majority voting). As the last period depicts a different situation where sustaining the resource is no longer required, the following results regarding the average percentage taken and profits as well as analyses of the voting proposals are all based on periods one to five.

Extraction behaviour differed depending on whether group members had asymmetric or symmetric opportunities to extract from the resource. Participants in the symmetric condition took across choice systems on average 13.20% of the resource available (*SD* = 2.69); this was increased significantly to 14.24% in the asymmetric condition (*SD* = 4.36, Cohen’s *d* = 0.28). Advantaged group members across choice systems took a significantly higher percentage of the resource available (*M* = 15.58%, *SD* = 5.16) than did disadvantaged group members (*M* = 12.90%, *SD* = 2.81, Cohen’s *d* = 0.65, see Table 1).

**Table 1:** General and differential effects of choice systems and asymmetry on average percentage taken with random effects for person (*N* = 248).

	Fixed effects						Random effects
	*B*	*95% CI*	*SE*	*df*	*t*	*p*	*SD*
Intercept	13.89	13.57, 14.21	0.16	496	85.53	< 0.001	1.73
Asymmetry	1.04	0.40, 1.68	0.32	245	3.20	0.002	
Advantaged	2.68	1.85, 3.51	0.42	245	6.38	< 0.001	
Intercept	14.46	13.81, 15.11	0.33	494	43.78	< 0.001	5.20
Median	− 1.14	− 1.80, − 0.48	0.34	494	− 3.39	< 0.001	5.29
MVoting	− 0.85	− 1.38, − 0.32	0.27	494	− 3.14	0.002	4.25
Intercept	14.59	13.98, 15.20	0.31	490	47.19	< 0.001	4.80
Median	− 1.23	− 1.85, − 0.60	0.32	490	− 3.87	< 0.001	4.92
MVoting	− 0.87	− 1.40, − 0.34	0.27	490	− 3.21	0.001	4.19
Asymmetry	1.45	0.24, 2.67	0.62	245	2.36	0.019	
Advantaged	5.06	3.49, 6.64	0.80	245	6.33	< 0.001	
Median × Asym	− 1.03	− 2.27, 0.21	0.63	490	− 1.62	0.105	
Median × Adv	− 5.06	− 6.67, − 3.45	0.82	490	− 6.18	< 0.001	
MVoting × Asym	− 0.22	− 1.27, 0.84	0.54	490	− 0.40	0.688	
MVoting × Adv	− 2.09	− 3.46, − 0.72	0.70	490	− 2.99	0.003	

Median and MVoting are dummy coded with individual choice as reference; Asymmetry is contrast coded to compare symmetric versus asymmetric groups; Advantaged is contrast coded to compare advantaged versus disadvantaged group members; all tests are two-sided.

*MVoting* Majority voting, *Asym* Asymmetry, *Adv* Advantaged.

As we predicted, extraction behaviour also differed across choice systems. Participants took on average 14.46% of the resource (*SD* = 5.20) in the individual choice system, and they took significantly less in the median choice system (*M* = 13.32%, *SD* = 1.49, Cohen’s *d* = 0.22) and in the majority voting system (*M* = 13.62%, *SD* = 3.62, Cohen’s *d* = 0.21; see Table 1). The results were robust when applying a non-parametric test. A Wilcoxon signed rank test indicated that the median of differences in the average percentage taken between individual choice and median choice (*Z* = − 2.47, *p* = 0.013), as well as individual choice and majority voting (*Z* = − 3.03, *p* = 0.002) was different from zero in the expected direction. Whereas the average percentage taken decreased under median choice and majority voting for the majority of participants, we also observed cases where the average percentage taken did not change or even increased, which is reflected in the random effects for person (see Table 1). The effects of the choice systems on average percentage taken did not depend on the order of systems (see Supplementary Information).

**Figure 2:**
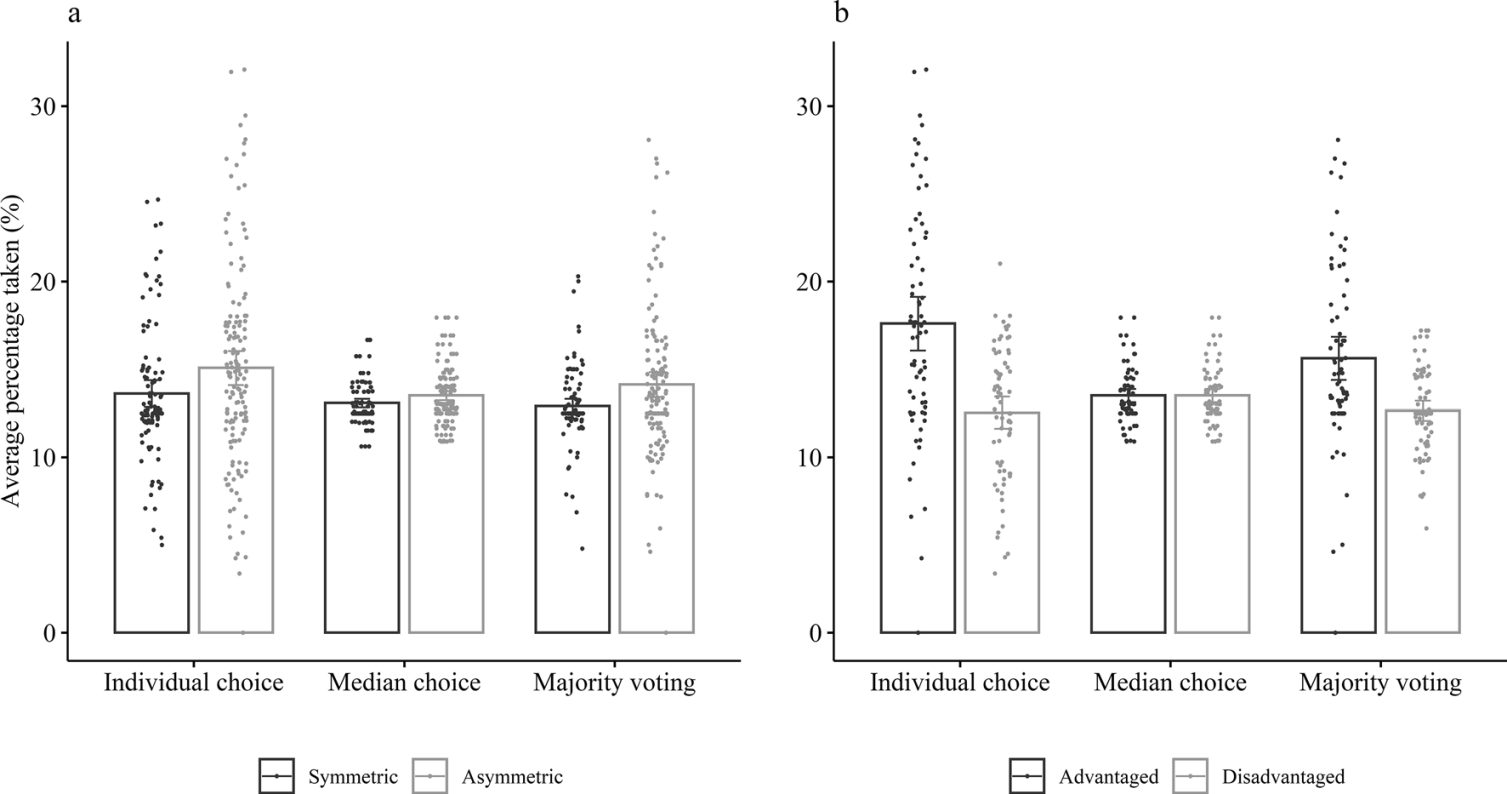
Average percentage taken across first five periods depending on choice system and (**a**) symmetric versus asymmetric groups, and (**b**) advantaged versus disadvantaged group members within asymmetric groups; error bars represent 95% confidence intervals.

The analysis examining the interplay of asymmetric opportunities and collective choice on extraction behaviour revealed no evidence that the collective choice systems had differential effects on symmetric versus asymmetric groups (see Table 1 and Fig. 2a). However, there was an interaction between both collective choice systems and the contrast between advantaged and disadvantaged group members, indicating differential effects of collective choice for advantaged and disadvantaged group members (see Table 1 and Fig. 2b). In the median choice system, this was characterised by a large decrease in the average percentage taken by advantaged group members (*b* = − 4.10, *t*(490) =  − 7.08, *p* < 0.001), and a small *increase* for disadvantaged group members (*b* = 0.96, *t*(490) = 1.66, *p* = 0.097; the conditional effects describe the difference in means between two conditions under a certain condition: here, difference between average percentage taken in the median choice system and the individual choice system by disadvantaged group members). In the majority voting system, advantaged group members’ extraction level decreased (*b* = − 1.98, *t*(490) =  − 4.02, *p* < 0.001), whereas the average percentage taken did not change for disadvantaged group members (*b* = 0.11, *t*(490) = 0.22, *p* = 0.828). Therefore, collective choice mainly reduced over-consumption by advantaged group members, which fostered the overall lower percentage taken. The median choice by its nature eliminated the difference in extraction levels between advantaged and disadvantaged group members, yet advantaged group members still extracted significantly more than disadvantaged group members in the majority voting system (*b* = 2.97, *t*(245) = 5.25, *p* < 0.001). In sum, median choice, as well as majority voting, fostered sustainable resource management in comparison to individual choice in both symmetric and asymmetric groups.

#### Characteristics of the majority voting system

Analysing the voting proposals over periods one to five revealed that in symmetric groups elected proposals were on average below the fair and sustainable rate of 12.5% (*M* = 11.99, *SD* = 1.80) and stipulated mostly equal extraction rates for all group members (with an average variation between group members of *M* = 0.10, *SD* = 0.45). Elected proposals in asymmetric groups were equally sustainable (*M* = 11.76, *SD* = 4.46), but varied significantly more between group members (*M* = 0.69, *SD* = 1.73). In symmetric and asymmetric groups, proposals that were not elected were on average also close to the sustainable rate (symmetric: *M* = 12.73, *SD* = 4.44; asymmetric: *M* = 12.51, *SD* = 3.74), but proposed extraction rates differed substantially between group members (symmetric: *M* = 1.33, *SD* = 2.47; asymmetric: *M* = 2.49, *SD* = 2.91; *t*(210) = -2.98, *p* = 0.003). In asymmetric groups, the variation in proposed extraction levels between group members was reflected in higher proposed extraction levels for advantaged and lower proposed extraction levels for disadvantaged group members (see Figure S1 in the Supplementary Information). Overall, a proposal was less likely elected the higher the average extraction rate (*b* = − 0.10, *z* = − 2.82, *p* = 0.005; odds ratio (*OR*) = 0.90), the more the average extraction rate deviated from the sustainable rate of 12.50 in either direction (*b* = − 0.44, *z* = − 10.79, *p* < 0.001; *OR* = 0.64), and the higher the variation among group members (*b* = − 0.75, *z* = − 7.70, *p* < 0.001; *OR* = 0.47).

To further explore the effect of the majority voting system on sustainable resource use, we examined the impact of successful voting decisions which ranged over periods one to five from 0 (participants extracted individually in all five periods) to 5 (participants reached a common decision in all five periods) with a mean of 2.69 (*SD* = 1.68). In periods with a successful voting decision, the average percentage taken was 11.86% (*SD* = 2.84) compared to 15.42% (*SD* = 5.81, Cohen’s *d* = 0.73) after unsuccessful voting (i.e., individual choice) decisions. After a successful voting decision, the average percentage taken was thus decreased compared to extraction under individual choice (*b* = − 2.58, *t*(470) = − 6.67, *p* < 0.001), whereas the average percentage taken was even slightly increased compared to extractions under individual choice when no proposal was elected (*b* = 0.87, *t*(434) = 2.51, *p* = 0.012; for more details see Supplementary Information). After successful voting decisions, there were no differences in the average percentage taken between symmetric and asymmetric groups (*b* = − 0.23, *t*(245) =  − 0.47, *p* = 0.638) or between advantaged and disadvantaged group members (*b* = 0.61, *t*(246) = 0.91, *p* = 0.364). If no proposal was elected, the average percentage taken differed between symmetric and asymmetric groups (*b* = 1.66, *t*(245) = 2.16, *p* = 0.032) and between advantaged and disadvantaged group members (*b* = 5.36, *t*(142) = 5.58, *p* < 0.001).

### Satisfaction and fairness ratings

Individuals in groups with an asymmetric distribution of opportunities across all choice systems rated satisfaction and fairness significantly lower (*M* = 4.48, *SD* = 1.22; possible range 1–7) than individuals in groups with a symmetric distribution of opportunities (*M* = 4.91, *SD* = 1.33, Cohen’s *d* = 0.34; see Table 2). Within asymmetric groups, advantaged group members reported overall higher satisfaction and fairness levels (*M* = 4.61, *SD* = 1.13) compared to disadvantaged group members (*M* = 4.34, *SD* = 1.28, Cohen’s *d* = 0.22). Satisfaction and fairness ratings were significantly increased in both collective choice systems (median choice: *M* = 5.03, *SD* = 1.13, Cohen’s *d* = 0.67; majority voting: *M* = 4.82, *SD* = 1.33, Cohen’s *d* = 0.49) compared to individual choice (*M* = 4.12, *SD* = 1.19; see Table 2). The results were robust when applying a non-parametric test. A Wilcoxon signed rank test indicated that the median of differences in satisfaction and fairness ratings between individual choice and median choice (*Z* = 9.12, *p* < 0.001), as well as individual choice and majority voting (*Z* = 6.89, *p* < 0.001) was different from zero in the expected direction.

**Table 2:** General and differential effects of choice systems and asymmetry on satisfaction and fairness ratings with random effects for person and group (*N* = 244).

Predictors	Fixed effects						Random effects	
	*B*	*95% CI*	*SE*	*df*	*t*	*p*	*SD *Person	*SD *Group
Intercept	4.62	4.47, 4.77	0.08	492	60.66	< 0.001	0.46	0.44
Asymmetry	− 0.43	− 0.73, − 0.12	0.15	60	− 2.81	0.006		
Advantaged	0.28	0.02, 0.54	0.13	185	2.14	0.033		
Intercept	4.12	3.91, 4.33	0.11	490	38.52	< 0.001	0.97	0.69
Median	0.91	0.71, 1.12	0.10	490	8.78	< 0.001	1.25	0.53
MVoting	0.70	0.44, 0.95	0.13	490	5.40	< 0.001	1.20	0.82
Intercept	4.11	3.89, 4.32	0.11	486	37.72	< 0.001	0.94	0.70
Median	0.90	0.69, 1.11	0.11	486	8.48	< 0.001	1.23	0.55
MVoting	0.65	0.41, 0.90	0.13	486	5.17	< 0.001	1.19	0.78
Asymmetry	− 0.19	− 0.63, 0.24	0.22	60	− 0.88	0.384		
Advantaged	0.58	0.27, 0.89	0.16	185	3.69	< 0.001		
Median × Asym	− 0.14	− 0.56, 0.28	0.21	486	− 0.66	0.507		
Median × Adv	− 0.51	− 0.92, 0.11	0.21	486	− 2.50	0.013		
MVoting × Asym	− 0.59	− 1.09, − 0.09	0.25	486	− 2.33	0.020		
MVoting × Adv	− 0.39	− 0.77, 0.002	0.20	486	− 1.95	0.051		

Median and MVoting are dummy coded with individual choice as reference; Asymmetry is contrast coded to compare symmetric versus asymmetric groups; Advantaged is contrast coded to compare advantaged versus disadvantaged group members; all tests are two-sided; due to a technical error the evaluation of the majority voting system was missing for one group: Estimates are therefore based on *N* = 244.

*MVoting* Majority voting, *Asym* Asymmetry, *Adv* Advantaged.

**Figure 3:**
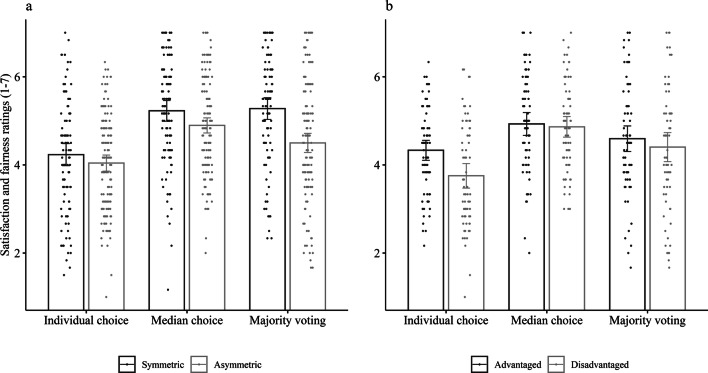
Satisfaction and fairness ratings depending on choice system and (**a**) symmetric versus asymmetric groups, and (**b**) advantaged versus disadvantaged group members within asymmetric groups; error bars represent 95% confidence intervals.

The analysis including the interaction of the choice systems and the asymmetry contrasts revealed no interaction of asymmetry with median choice (see Table 2 and Fig. 3a). However, asymmetry moderated the effect of majority voting on satisfaction and fairness indicating that satisfaction and fairness ratings increased significantly in symmetric groups (*b* = 1.05, *t*(486) = 7.46, *p* < 0.001) but only to a lesser extent in asymmetric groups (*b* = 0.46, *t*(486) = 3.88, *p* < 0.001). In an attempt to further explore this pattern, we tested whether asymmetric opportunities lead to fewer successful voting decisions, and whether this in turn predicts satisfaction and fairness ratings. We indeed found that for the majority voting system the number of successful voting decisions (how often a proposal was elected ranging from 0 to 5) partially mediated the effect of asymmetry on satisfaction and fairness ratings (indirect effect = − 0.43 [− 0.62, − 0.26]). Asymmetry led to fewer successful voting decisions (*b* = − 1.15, *t* = − 5.56, *p* < 0.001), whereas the number of successful voting decisions predicted satisfaction and fairness ratings (*b* = 0.38, *t* = 8.14, *p* < 0.001). The order in which participants completed the choice systems had an impact on satisfaction and fairness ratings. Specifically, symmetric groups rated individual choice more favourably when it was the first system they experienced compared to when they first experienced one of the collective choice systems. Therefore, the increase in satisfaction and fairness ratings was larger in symmetric groups when they started with collective choice. For asymmetric groups, the increase in satisfaction and fairness ratings in the majority voting system was larger when they started with individual choice (see Supplementary Information for details).

The increase in satisfaction and fairness ratings in the median choice system depended on group members’ status as advantaged or disadvantaged, indicating a smaller increase for advantaged (*b* = 0.60, *t*(486) = 3.77, *p* < 0.001) than for disadvantaged (*b* = 1.11, *t*(486) = 7.02, *p* < 0.001) group members (see Fig. 3b). The increase in satisfaction in the majority voting system compared to individual choice was also smaller and non-significant for advantaged (*b* = 0.26, *t*(486) = 1.58, *p* = 0.114) compared to disadvantaged group members (*b* = 0.65, *t*(486) = 3.90, *p* < 0.001), although the interaction term did not reach significance (*p* = 0.051).

### Profits

**Figure 4:**
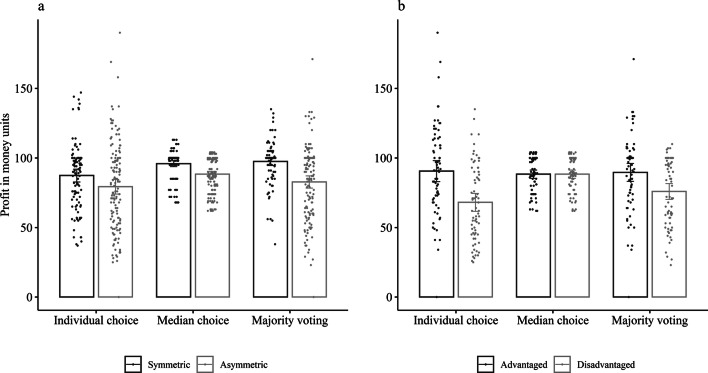
Profit in money units across first five periods depending on choice system and (**a**) symmetric versus asymmetric groups, and (**b**) advantaged versus disadvantaged group members within asymmetric groups; error bars represent 95% confidence intervals.

Although in both collective choice systems participants took on average a lower percentage of the resource, both median choice and majority voting led to overall higher profits compared to individual choice (see Table 3 and Fig. 4). That is, individuals took a lower percentage in each period and therefore sustained a larger resource to harvest from in the next periods. Across systems, asymmetric groups earned significantly less than symmetric groups, and advantaged group members overall earned more than disadvantaged group members. The effect of majority voting in increasing profits was larger for symmetric groups (*b* = 10.13, *t*(490) = 3.87, *p* < 0.001) and not significant for asymmetric groups (*b* = 3.43, *t*(490) = 1.54, *p* = 0.124), whereas the effect of median choice did not differ between symmetric and asymmetric groups (see Table 3 and Fig. 4a). The increase in profits depended on the order of the choice systems, in that symmetric groups earned significantly more in the collective choice systems compared to individual choice when they started with one of the collective choice systems, whereas asymmetric groups earned significantly more in the collective choice systems in comparison to individual choice when they started with individual choice (see Supplementary Information). The effects of median choice and majority voting differed for advantaged versus disadvantaged group members, indicating no effect for the former (median choice: *b* = − 2.24, *t*(490) =  − 0.68, *p* = 0.494; majority voting: *b* = -0.96, *t*(490) =  − 0.30, *p* = 0.761) but a large increase in profit for the latter (median choice: *b* = 20.25, *t*(490) = 6.19, *p* < 0.001; majority voting: *b* = 7.82, *t*(490) = 2.48, *p* = 0.013; see Fig. 4b). Both collective choice systems therefore increased the payoff for symmetric groups and disadvantaged group members, whereas there was no effect for advantaged group members who profited equally from all three systems.

**Table 3:** General and differential effects of choice systems and asymmetry on profits with random effects for person (*N* = 248).

	Fixed effects						Random effects
	*B*	*95% CI*	*SE*	*df*	*t*	*p*	*SD*
Intercept	86.81	84.87, 88.76	0.99	496	87.73	< 0.001	10.61
Asymmetry	− 10.10	− 13.99, − 6.21	1.98	245	− 5.11	< 0.001	
Advantaged	12.06	7.03, 17.10	2.56	245	4.72	< 0.001	
Intercept	82.67	79.07, 86.26	1.83	494	45.20	< 0.001	2.88
Median	8.80	5.19, 12.41	1.84	494	4.79	< 0.001	2.89
MVoting	6.24	2.87, 9.62	1.72	494	3.64	< 0.001	2.70
Intercept	81.97	78.52, 85.43	1.76	490	46.56	< 0.001	2.73
Median	8.84	5.33, 12.36	1.79	490	4.95	< 0.001	2.77
MVoting	5.67	2.28, 9.05	1.72	490	3.29	0.001	2.67
Asymmetry	− 8.03	− 14.95, − 1.11	3.51	245	− 2.28	0.023	
Advantaged	22.49	13.52, 31.45	4.55	245	4.94	< 0.001	
Median × Asym	0.49	− 6.53, 7.50	3.57	490	0.14	0.891	
Median × Adv	− 22.49	− 31.57, − 13.40	4.62	490	− 4.86	< 0.001	
MVoting × Asym	− 6.70	− 13.46, 0.05	3.44	490	− 1.95	0.052	
MVoting × Adv	− 8.78	− 17.53, − 0.02	4.46	490	− 1.97	0.049	

Median and MVoting are dummy coded with individual choice as reference; Asymmetry is contrast coded to compare symmetric versus asymmetric groups; Advantaged is contrast coded to compare advantaged versus disadvantaged group members; all tests are two-sided.

*MVoting* Majority voting, *Asym* Asymmetry, *Adv* Advantaged.

## Discussion

In the present investigation, we examined whether collective choice can foster sustainable resource management in the presence of asymmetric opportunities among actors involved in the decision. We found that an asymmetric distribution of opportunities was associated with more unsustainable resource management. Both of our implementations of collective choice, median choice and majority voting, increased sustainable resource management in comparison to individual choice. Although we did not find evidence that the decrease in the average percentage taken in the collective choice systems was statistically different for symmetric and asymmetric groups, the effect was larger for asymmetric groups on a descriptive level. The collective choice systems were effective in promoting sustainable behaviour in asymmetric groups as they restricted the extraction behaviour of advantaged group members who tended to overuse the common resource under individual choice. Symmetric groups, on the other hand, were already close to a sustainable extraction level in the individual choice condition, which could explain the weaker effect of collective choice in these groups.

Our findings highlight the importance of taking into consideration asymmetric distributions of opportunities among actors in social dilemma situations. In line with other findings^4–10^, we show that asymmetry impedes cooperation and fosters unsustainable resource management. This was driven by advantaged group members exploiting their privileged position to overuse common resources. Over-consumption by advantaged group members was reduced in the collective choice conditions, which supports the notion that collective choice can foster sustainable resource management in the presence of an asymmetric distribution of opportunities. The implemented median choice system completely eliminates differences in extraction behaviour and profit between advantaged and disadvantaged group members, whereas the implemented majority voting system allows for differences in extraction behaviour; this resulted in the preservation of higher extraction rates and profits of advantaged compared to disadvantaged group members. Overall, both collective choice systems improved resource management and collective welfare in symmetric as well as asymmetric groups and should therefore be considered as solutions to common resource dilemmas involving asymmetric distribution of opportunities among actors. Although the average percentage taken differed only by a few percentage points between choice systems and asymmetry conditions, this has an enormous impact on resource development. For example, with the average percentage taken under individual choice (14.46%) the resource would decrease to contain only 60 units after period six, whereas with the average percentage taken under median choice (13.32%) the resource would still contain 109 units. If every group member extracted as advantaged group members did under individual choice (17.44%), the resource would decrease to only 9 units within six periods, whereas if every group member extracted as disadvantaged group members did under individual choice (12.92%), it would still contain 130 units, which is fourteen times the amount.

Relevant to our second outcome, satisfaction and fairness ratings, both collective choice systems were evaluated more favourably compared to individual choice. However, satisfaction and fairness ratings in the majority voting system compared to individual choice increased only in symmetric groups, which might be explained by a lower rate of successful voting decisions in asymmetric groups. The increase in satisfaction and fairness ratings in the collective choice systems did not depend on whether group members were advantaged or disadvantaged. Although advantaged individuals extracted more under individual choice, they did not rate this system more favourably than the collective choice systems. These findings suggest that many individuals involved in resource dilemmas support a shift from individual to collective choice, even if that might restrict their own extraction behaviour as advantaged group members. It would have been an interesting additional dependent variable to ask participants directly which system they prefer for another round of play. With regard to different punishment systems, it has been shown that outcome satisfaction and fairness perception predicted the willingness to continue under the present system^34^. Accordingly, there is good reason to assume that implementation of collective choice systems will find support among the involved actors.

Overall, profits were increased in both collective choice systems compared to individual choice. This was characterised by disadvantaged individuals and symmetric groups gaining significantly under the collective choice systems, while advantaged individuals profited equally from all three systems. Thus, collective choice fosters sustainable resource management and improves the welfare of the collective. Notably, however, in both collective choice systems profit as well as satisfaction and fairness ratings were still significantly lower in asymmetric compared to symmetric groups. This implies that research should not focus just on (collective) choice systems to foster sustainable resource management, but also search for solutions to reduce asymmetry between actors. In the following section, we outline additional ideas for future research along with limitations of our study.

In the majority voting system, a voting decision was only reached in half of the trials, which is comparable to rates observed in other studies^17,21^. In this study, we aimed to examine whether installing a majority voting system increases sustainable resource use without the requirement of reaching a successful voting decision. Previous research suggests that the process of deciding as a group can increase cooperation even after unsuccessful voting decisions^17^. However, extraction behaviour depended on whether or not groups reached an agreement^21,35^, that is, extraction levels after successful voting decisions were substantially decreased compared to individual choice while extraction levels after unsuccessful voting decisions were even slightly increased compared to extractions under individual choice. Although the majority voting system overall improved sustainable resource management, it still resulted in differences in extraction level and profit between advantaged and disadvantaged group members. In fact, the variation in proposed extraction levels between group members in asymmetric groups reflected overall higher proposed extraction levels for advantaged and lower proposed extraction levels for disadvantaged group members, whereas there were on average no differences in proposed extraction levels for group members of symmetric groups. Voting proposals that were supported by a majority in symmetric and asymmetric groups mainly suggested equal extraction rates for all group members and were on average sustainable (*M* = 11.86%; the average extraction after a successful voting decision was even below the sustainable rate of 12.5%, allowing the resource to (re)grow). In asymmetric groups the variation in elected voting proposals was increased compared to symmetric groups, but still small compared to proposals that were not elected. Therefore, the differences in extraction levels among group members originated mainly from unsuccessful voting decisions followed by individual extraction.

Whether or not a common decision was reached in the majority system also affected satisfaction and fairness ratings. Asymmetric groups often failed to find agreement in the majority voting system, which in turn predicted lower satisfaction and fairness ratings. It is important to interpret these results with caution, as the differences between extraction after successful and unsuccessful voting decisions could be due to the decision process via majority voting but could also (at least partially) be explained by the fact that more cooperative groups more often reached a common decision. Of note, groups contributed unequal numbers of observations regarding the extraction after successful and unsuccessful voting decisions (some groups always reached a common decision and some never did). Therefore, it is an interesting topic for future research to explore how to increase the rate of successful voting decisions, for instance by allowing more than one preference or voting round to answer the question about whether reaching a common decision via majority voting per se can increase sustainable behaviour.

Regarding the generalisability of the results, some specificities of the implemented experimental conditions should be considered. The first concerns the manipulation of asymmetry: In the present study, we tested the effect of artificially and randomly created differences in opportunity to access a common resource. Even in this setting, we find that advantaged group members overuse the common resource. This effect could be even stronger when differences in opportunities are established based on prior efforts^25, 36^. It remains an open question to what extent the findings with this manipulation of asymmetry can be generalised to contexts where inequality reflects meritocratic or other social rules. It is possible that advantaged group members who feel they earned their status would evaluate collective choice systems that restrict their extraction behaviour more negatively than advantaged group members who were randomly assigned to this role. Another characteristic of our manipulation of asymmetry are the equal rates of advantaged and disadvantaged group members within asymmetric groups. This precludes that members of one group determine which proposal is elected in the majority voting system. It remains possible that proposals with more variation in extraction levels among group members would be elected if one group (advantaged or disadvantaged) constituted a majority. Varying the group composition regarding advantaged and disadvantaged group members is therefore yet another important entry on the agenda for future research in this field.

A similar rationale applies to the collective choice systems, as we implemented two specific forms of median choice and majority voting. It may prove useful for future research to vary single aspects of the choice systems to provide information on what specific aspects of the choice systems foster sustainable resource management (e.g., voting on a proposal versus proposing extraction levels for each group member). Also, the study design included a fixed number of six periods under each choice system. Our data suggests that knowing the common resource game would end after six periods influenced behaviour, particularly in the sixth and therefore last period, in which many participants exploited the resource completely. It poses an interesting question whether the definite ending after six periods also influenced behaviour in the periods before and participants would have extracted more sustainably if the number of periods was undisclosed. Also, there may be individual differences in how the resource game was construed (as finitely or indefinitely repeated). However, as the resource size directly determined the maximum extraction possibilities, we suppose that differences in how the resource game was construed (if such differences were actually given) would play out primarily in the sixth period. Another interesting topic for future research would be to examine the variability in extraction levels and the effects of the collective choice systems between individuals (random effects) including potentially underlying characteristics (e.g., inequality aversion). It should also be noted that we used a student sample (mostly other than psychology students), recruited from a western university, and thus should be cautious with generalisations to populations with more diverse backgrounds^37^. It is possible that these participants considered the level of incentives to be rather low, however there is evidence from other economic games showing that behaviour under high and low stakes is comparable^38,39^.

To conclude, the present investigation demonstrates that asymmetric opportunities within groups foster unsustainable resource management. Both implemented collective choice systems, median choice and majority voting, increase sustainable resource management in the presence of asymmetry, as they restrict the over-consumption behaviour of advantaged group members. This leads to overall higher profits as reflected in an increase for disadvantaged group members, whereas advantaged group members profit equally from all three systems. In addition, both collective choice systems are evaluated more favourably than individual choice independent of asymmetry. Thus, we identified collective choice as an effective means to overcome a social dilemma and to foster sustainable resource management in the presence of asymmetric opportunities.

## Method

It is our understanding that only approbated psychologists conducting clinical studies are legally obligated to obtain ethical oversight (according to the Declaration of Helsinki). For psychological studies without any clinical measures, the American Psychological Association (APA) and the German Association of Psychologists (DGPs) provide guidelines for the responsible treatment of participants. We conducted our study in full accordance with these guidelines. The implementation of economic games to study behaviour in social dilemma situations was approved by the University of Ulm’s Central University Research Ethics Committee (application number 297/16). The implementation of follow-up studies applying methods that have been approved before does not, at our institute, require completion of an entire approval process. There was no deception of participants. Participants gave informed consent before starting the study protocol, and the study was conducted in accordance with relevant guidelines and regulations.

### Common resource game

#### Basic structure of the resource game

Participants played a total of 18 periods of a common resource game in groups of four. In the first period of each choice system the resource contained 160 units. After the group members made their extraction decision, the remaining units were doubled for the next period. Thus, if groups took exactly half of the units available, the resource would remain the same. If they took more than half of the units, the resource would decrease. If they took less than half of the units, the resource would increase. The resource could, however, never exceed the original number of 160 units. Participants were always informed about how many units the resource contained and the extraction limit before making their decision. All interactions were computer-mediated using z-Tree^40^. Decisions in the resource game were incentivised, as participants’ extracted units were transformed into real money at the end of the experiment (for every unit extracted they received 0.01€). Earnings from the common resource game ranged between 1.24€ and 5.30€. When informed about their final income at the end of the experiment, participants were told that they would receive a minimum of 3€ even if their earnings fell below that.

#### Choice systems

In the individual choice system, participants decided how many units to extract from the resource individually and simultaneously. After they made their decision, they were informed how many units the group extracted in total and how many units were left for the next period. In the *median choice system*, each group member indicated how many units he or she wanted to extract. The median of those proposals was extracted for each group member^14^. For instance, if the four group members wanted to extract 10, 12, 12, and 16 units, the median of 12 units would be automatically extracted for each group member. If they wanted to extract 10, 12, 14 and 16 units, the mean of the two middle proposals would be computed. The median was automatically extracted for all group members, independent of their status as advantaged or disadvantaged. Thus, the extraction in the median system could exceed the extraction limit for disadvantaged group members (this occurred in only 3.5% of trials). After making their decision, participants were informed about the proposals of each group member, the median, and how many units were left for the next period. In the *majority voting system*, participants were asked to propose how many units each group member (including themselves) should extract. They were then presented the four proposals and asked to vote on which one should be implemented. Each participant was granted a single vote and no one could not abstain from voting. Votes for identical proposals were combined. If one proposal received a majority (at least three of four votes), it was implemented. If no proposal received a majority, each group member would decide individually how much to extract in that period. After voting, participants were informed if a proposal received a majority, and if so, how many units each group member received and how many units the resource contained for the next period. If no proposal received a majority, participants were informed that they could extract individually in that period, about the extraction decision of all group members, and how many units the resource contained for the next period.

### Asymmetry conditions

Participants were randomly assigned to one of two asymmetry conditions. In symmetric groups, all group members could extract up to one fourth (25%) of the resource in each period. In asymmetric groups, two advantaged group members could extract up to one third (33%) of the resource, while two disadvantaged group members could only extract up to one sixth (17%) of the resource. The asymmetry condition and the role assignment as advantaged (*n* = 72) or disadvantaged (*n* = 72) within asymmetric groups was constant over the three choice systems.

### Participants and procedure

A priori power analyses in multi-level settings require detailed information (e.g., parameter estimates for fixed and random effects from a pilot study) that was not available. We therefore planned on including at least 220 participants in the study. Within time and budget restrictions, we were able to recruit a total of 248 participants on campus of a German university (*M*_age_ = 21.34, *SD* = 3.25; these statistics are based on *N* = 244 since due to a technical error data concerning demographic information was not saved for one group). After giving informed consent, participants read the instructions for the common resource game including information on the basic structure, the choice systems, and the asymmetry conditions. The order of the systems was randomised across groups. Participants were then informed if their group was assigned to the symmetric (*n* = 104) or asymmetric condition (*n* = 144) and what percentage of the resource they could extract in each period.

For exploratory purposes, we first measured participants’ social value orientation (SVO) using the slider measure^41^, as SVO is a strong predictor of cooperation^42^. Participants were asked to allocate resources between themselves and another person on a predefined continuum of joint payoffs. Decision making was incentivised as one of the allocation decisions was randomly selected and paid out to the participants. SVO angles (for calculation details see^41^) ranged between − 16.26 and 61.39 with a mean of 27.15 (*SD* = 15.49). Therefore, the vast majority of participants (*N* = 164) was characterised as prosocial, whereas 79 could be described as individualistic, 3 as altruistic, and 2 as competitive. Due to power issues, however, this measure was not included in any of the analyses for the present contribution.

Participants sat in cubicles to ensure privacy and anonymity. The number assigned to each group member was randomly shuffled after each period: The participant who appeared as “group member 1” in period 1 could be “group member 3” in period 2. Therefore, participants could not deduce behaviour of a specific person across periods to prevent that previous behaviour of specific group members influenced later decisions (i.e., the voting proposals). Note that despite this procedure, advantaged group members could infer something of the behaviour of other advantaged group members in cases where the other extracted more than one sixth of the resource. Additionally, to prevent order effects, the numbers were shifted for each group member, so that participant 1 would see the decision of participant 2 as “group member 1,” the decisions of participant 3 as “group member 2,” and the decisions of participant 4 as “group member 3.” For participant 2, the decisions of participant 3 were displayed as “group member 1,” the decisions of participant 4 as “group member 2,” and the decisions of participant 1 as “group member 3.”

After each system, we assessed participants’ outcome satisfaction and their perception of fairness by three items^34^ (outcome satisfaction: “In the last six periods I was satisfied with my experiences.”; “In the last six periods I was satisfied with my income.”; “In the last six periods I was satisfied with the decision over the number of units I extracted.”; fairness perception: “In the last six periods I had the opportunity to influence the group result.”; “In the last 6 periods, the process deciding over the extraction of each group member was fair.”; “In the last six periods personal motives of other group members (e.g., greed) influenced my income.”) Participants responded on a scale ranging from 1 = *I strongly disagree* to 7 = *I strongly agree*. After all 18 periods were finished, we assessed demographic information (age, sex, native language) before informing participants about their final income. Participants received a minimum of 3€ (≈ $3.40) plus the amount they earned beyond that in the common resource game and the SVO measure (*M* = 4.00€ ≈ $4.50, *SD* = 0.43).

### Variables and statistical analysis

The first dependent variable was extraction behaviour. To account for the changing number of units available, we calculated the percentage taken by each individual relative to the size of the resource available. For the regression analyses, we averaged the percentage taken over all periods within one system. As participants behaved differently in the sixth period (as it was the last period, they took significantly more than in the first five periods and many groups depleted the resource completely), we calculated the average percentage taken across periods one to five as the central dependent variable. The second outcome was the evaluation reflecting outcome satisfaction and fairness perception. Principal axis factor analyses revealed that both scales were represented by a common factor; we therefore combined all six items into one measure of satisfaction and fairness (Cronbach’s α between 0.71 and 0.82; similar results occurred when examining the two constructs separately: see Supplementary Information for more details). To compare the choice systems, we used dummy variables for median choice and majority voting with individual choice as reference group. To examine the moderating role of asymmetry, we contrast coded the conditions to compare symmetric versus asymmetric groups on the one hand and (within asymmetric groups) advantaged versus disadvantaged group members on the other.

To account for the nested structure of the data, we applied multilevel mixed regressions using the R package nlme^43^. For the average percentage taken, the intra class correlation (ICC) indicated that a substantial proportion of variance was explained by the participants as level-2 units (ICC = 0.25), and only a small percentage was additionally explained by the groups as level-3 units (ICC = 0.05). For satisfaction and fairness ratings, there was substantial variance due to participants (ICC = 0.14) and groups (ICC = 0.14). We therefore included random effects for participants for the average percentage taken and random effects for participants and groups for satisfaction and fairness ratings in the regression analyses. The fixed effects describe the average effect of choice systems across participants, whereas the random slopes indicate the variation in intercept and slope between participants.

## Supplementary information

Supplementary information.

## Data Availability

All data files, instructions for the common resource game, and satisfaction and fairness measures are available on the Open Science Framework (OSF, see https://osf.io/hu85y/).

## Code availability

The corresponding SPSS and R code is also available on the OSF: https://osf.io/hu85y/.
