# Supplementary material for: Collective choice fosters sustainable resource management in the presence of asymmetric opportunities
Source: Sci Rep. 2020 Jul 1;10:10724. doi: 10.1038/s41598-020-67757-1 (PMC7329894; doi:10.1038/s41598-020-67757-1)
Supplement: Supplementary file 1 — Supplementary information. [file 41598_2020_67757_MOESM1_ESM.pdf]

Supplementary Information

Collective choice fosters sustainable resource management in the presence of asymmetric opportunities

Laila Nockur[1*], Laetitia Arndt[1], Johannes Keller[1], & Stefan Pfattheicher[2]

[1] Ulm University, Albert-Einstein-Allee 47, 89069 Ulm, Germany; [2]Aarhus University, Bartholins Allé 11, 8000 Aarhus C, Denmark

Corresponding author:
Laila Nockur
Ulm University
Department of Social Psychology
Albert-Einstein-Allee 47
89069 Ulm, Germany
laila.nockur@uni-ulm.de

Time trends in average percentage taken

Extraction behaviour over the six periods followed a U-shaped trend, characterised by larger extraction rates in the first period and increasing extraction rates in periods five and six compared to extraction rates during the mid-periods (see Table S1). Being the last, period six constitutes a different situation where sustaining the resource is no longer required. This is reflected in a substantial increase in extraction with around 50% of the participants extracting (or respectively proposing to extract) the maximum in period six.

Table S1. Time trends in extraction levels over the six periods.

	Fixed effects						Random effects
Predictors	*B*	*95% CI*	*SE*	*df*	*t*	*p*	*SD Person*
Intercept	14.34	14.01, 14.67	0.19	4155	76.27	<.001	2.31
Period 1 vs 2	-1.13	-1.71, -0.55	0.30	4155	-3.82	<.001	
Period 2 vs 3	-0.13	-0.72, 0.45	0.30	4155	-0.45	.649	
Period 3 vs 4	0.40	-0.18, 0.98	0.30	4155	1.35	.178	
Period 4 vs 5	1.18	0.60, 1.76	0.30	4155	3.97	<.001	
Period 5 vs 6	2.64	2.06, 3.23	0.30	4155	8.86	<.001	

Average percentage taken after successful and unsuccessful voting decisions

In the majority voting system consumption behaviour differed substantially depending on whether a common decision was reached (i.e., the elected proposal was executed) or not (i.e., group members decided individually). After a common decision, the average percentage was decreased compared to individual extraction (see Table S2), whereas the average percentage taken was even slightly increased compared to extractions in the individual choice system when no common decision was reached (see Table S3).

Table S2. General and differential effects of choice systems and asymmetry on average percentage taken with random effects for person for behaviour after successful voting decisions.

	Fixed effects						Random effects
	B	*95% CI*	*SE*	*df*	*t*	*p*	*SD*
Intercept	13.33	13.04, 13.62	0.15	472	90.12	<.001	0.44
Asymmetry	0.65	0.07, 1.23	0.29	245	2.22	.027	
Advantaged	1.98	1.22, 2.73	0.39	245	5.13	<.001	
Intercept	14.46	13.81, 15.11	0.33	470	43.78	<.001	5.20
Median	-1.14	-1.80, -0.48	0.34	470	-3.39	<.001	5.29
MVoting	-2.58	-3.35, -1.82	0.39	470	-6.67	<.001	5.99
Intercept	14.59	13.98, 15.20	0.31	466	47.20	<.001	4.79
Median	-1.23	-1.85, -0.60	0.32	466	-3.87	<.001	4.92
MVoting	-2.76	-3.50, -2.01	0.38	466	-7.29	<.001	5.70
Asymmetry	1.45	0.24, 2.67	0.62	245	2.36	.019	
Advantaged	5.06	3.49, 6.64	0.80	245	6.34	<.001	
Median × Asym	-1.03	-2.27, 0.21	0.63	466	-1.63	.105	
Median × Adv	-5.06	-6.67, -3.45	0.82	466	-6.18	<.001	
MVoting × Asym	-1.69	-3.15, -0.22	0.75	466	-2.26	.024	
MVoting × Adv	-4.46	-6.40, -2.52	0.99	466	-4.52	<.001	

Note. MVoting = Majority voting, Asym = Asymmetry, Adv = Advantaged; Median and MVoting are dummy coded with individual choice as reference; Asymmetry is contrast coded to compare symmetric versus asymmetric groups; Advantaged is contrast coded to compare advantaged versus disadvantaged group members; all tests are two-sided.

After successful voting decisions, there were no differences in the average percentage taken between symmetric and asymmetric groups nor between advantaged and disadvantaged group members (see Table S2). If no proposal reached a majority, the average percentage

taken differed between symmetric and asymmetric groups and between advantaged and disadvantaged group members (see Table S3). It is important to interpret these results with caution, as groups contributed unequal observations regarding the extraction after successful and unsuccessful voting decisions (some groups always reached a common decision and some never did). Thus, the differences between extraction levels after successful and unsuccessful voting decisions could be due to the decision process via majority voting, but could also (at least partially) be explained by the fact that more cooperative groups were more likely to reach a common decision. Therefore, it remains a question for future research to examine whether reaching a common decision via majority voting per se can increase sustainable behaviour.

Table S3. General and differential effects of choice systems and asymmetry on average percentage taken with random effects for person for behaviour after unsuccessful voting decisions.

	Fixed effects						Random effects
	B	*95% CI*	*SE*	*df*	*t*	*p*	*SD*
Intercept	14.39	14.00, 14.77	0.20	436	73.68	<.001	1.97
Asymmetry	1.22	0.44, 1.99	0.39	245	3.10	.002	
Advantaged	3.37	2.38, 4.35	0.50	245	6.70	<.001	
Intercept	14.46	13.81, 15.11	0.33	434	43.78	<.001	5.20
Median	-1.14	-1.80, -0.48	0.34	434	-3.39	<.001	5.29
MVoting	0.87	0.19, 1.55	0.35	434	2.51	.012	4.84
Intercept	14.59	13.98, 15.20	0.31	430	47.19	<.001	4.80
Median	-1.23	-1.85, -0.60	0.32	430	-3.87	<.001	4.92
MVoting	0.86	0.18, 1.54	0.35	430	2.47	.014	4.86
Asymmetry	1.45	0.24, 2.67	0.62	245	2.36	.019	
Advantaged	5.06	3.49, 6.64	0.80	245	6.33	<.001	
Median × Asym	-1.03	-2.27, 0.22	0.63	430	-1.62	.105	
Median × Adv	-5.06	-6.67, -3.45	0.82	430	-6.18	<.001	
MVoting × Asym	0.20	-1.21, 1.62	0.72	430	0.28	.778	
MVoting × Adv	0.30	-1.42, 2.02	0.87	430	0.34	.731	

Note. MVoting = Majority voting, Asym = Asymmetry, Adv = Advantaged; Median and MVoting are dummy coded with individual choice as reference; Asymmetry is contrast coded to compare symmetric versus asymmetric groups; Advantaged is contrast coded to compare advantaged versus disadvantaged group members; all tests are two-sided.

Characteristics of voting proposals

Table S4. Characteristics of elected and rejected voting proposals over periods one to five.

		Average percentage taken			Average variation (*SD*) in percentage taken		
		Mean (SD)	*Min*	*Max*	*Mean (SD)*	*Min*	*Max*
Overall	Elected proposal	11.86 (3.48)	0.00	25.00	0.42 (1.34)	0.00	7.22
	Rejected proposal	12.55 (4.09)	0.00	25.00	2.05 (2.80)	0.00	12.94
	Individual extraction	15.42 (3.15)	10.24	23.91	6.14 (2.83)	0.00	12.07
Symmetric groups	Elected proposal	11.99 (1.80)	4.90	14.06	0.10 (0.45)	0.00	2.31
	Rejected proposal	12.85 (4.57)	0.00	25.00	1.33 (2.91)	0.00	12.50
	Individual extraction	14.31 (2.99)	10.36	21.88	5.03 (1.89)	0.00	8.49
Asymmetric groups	Elected proposal	11.76 (4.46)	0.00	25.00	0.69 (1.73)	0.00	7.22
	Rejected proposal	12.37 (3.77)	0.00	20.81	2.49 (2.91)	0.00	12.94
	Individual extraction	16.05 (3.08)	10.24	23.91	6.77 (3.08)	0.94	12.07

Note. Individual extraction refers to the extraction after unsuccessful voting decisions.

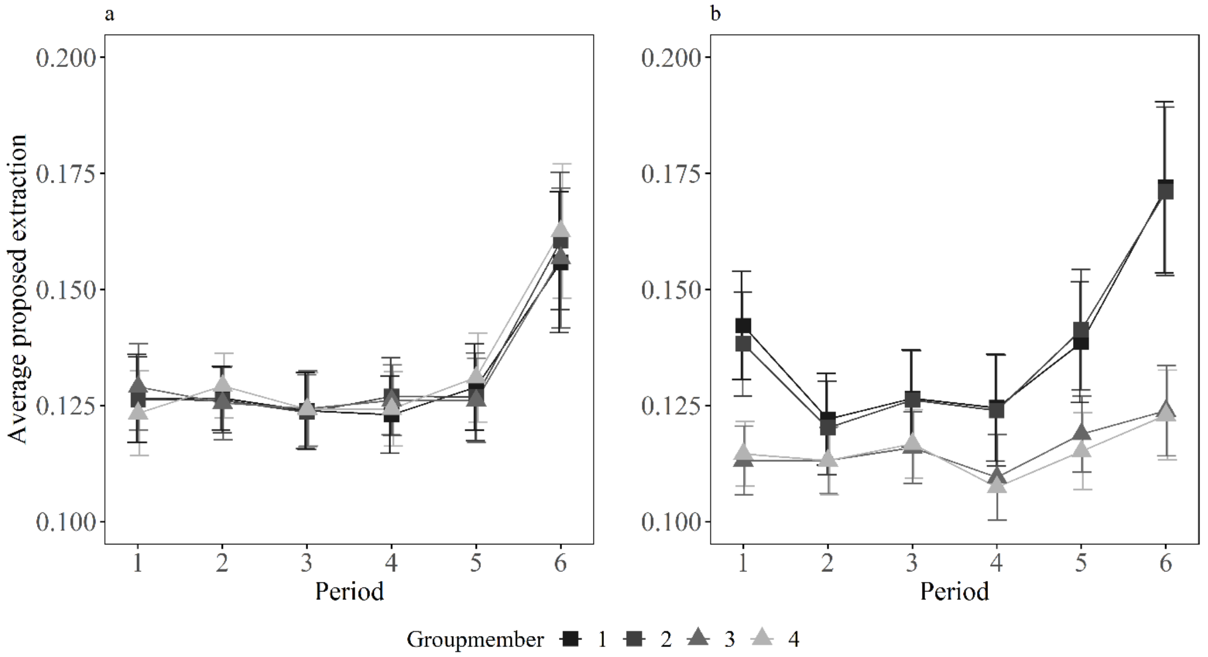

Figure S1. Average proposed extraction level over periods for each group member in (a) symmetric and (b) asymmetric groups. In asymmetric groups, group members 1 and 2 are advantaged and group members 3 and 4 are disadvantaged; error bars represent 95% confidence intervals.

Examining satisfaction and fairness ratings separately

Satisfaction ratings

Outcome satisfaction was measured by three items after six periods of the respective choice system. Internal reliability was acceptable (Cronbach's α between .75 and .81), therefore we computed the mean score of the three items as measure for outcome satisfaction. Asymmetric groups reported overall lower satisfaction; advantaged and disadvantaged group members did not differ in their overall satisfaction ratings (see Table S5). Both collective choice systems increased satisfaction ratings. However, the model including the choice systems and asymmetry contrasts revealed that majority voting increased satisfaction ratings only in symmetric groups (b = 0.78, t(486) = 4.94, p < .001) but not in asymmetric groups (b = 0.23, t(486) = 1.74, p = .082).

Table S5. General and differential effects of choice systems and asymmetry on satisfaction ratings with random effects for person and group (N = 244).

	Fixed effects						Random effects	
Predictors	*B*	*95% CI*	*SE*	*df*	*t*	*p*	*SD Person*	*SD Group*
Intercept	4.97	4.81, 5.13	0.08	492	61.30	<.001	0.73	0.39
Asymmetry	-0.45	-0.78, -0.13	0.16	60	-2.80	.007		
Advantaged	0.07	-0.25, 0.39	0.16	185	0.43	.670		
Intercept	4.66	4.44, 4.89	0.12	490	40.54	<.001	1.27	0.65
Median	0.57	0.36, 0.78	0.11	490	5.29	<.001	1.41	0.47
MVoting	0.46	0.20, 0.71	0.13	490	3.56	<.001	1.47	0.69
Intercept	4.65	4.42, 4.88	0.12	486	39.72	<.001	1.27	0.65
Median	0.55	0.34, 0.77	0.11	486	5.04	<.001	1.40	0.48
MVoting	0.42	0.17, 0.66	0.13	486	3.30	.001	1.46	0.64
Asymmetry	-0.21	-0.68, 0.25	0.23	60	-0.92	.362		
Advantaged	0.31	-0.10, 0.73	0.21	185	3.30	.137		
Median × Asym	-0.18	-0.61, 0.25	0.22	486	-0.81	.419		
Median × Adv	-0.42	-0.88, 0.04	0.23	486	-1.81	.071		
MVoting × Asym	-0.55	-1.05, -0.06	0.25	486	-2.19	.029		
MVoting × Adv	-0.31	-0.79, -0.16	0.24	486	-1.29	.197		

Note. MVoting = Majority voting, Asym = Asymmetry, Adv = Advantaged; Median and MVoting are dummy coded with individual choice as reference; Asymmetry is contrast coded to compare symmetric versus asymmetric groups; Advantaged is contrast coded to compare advantaged versus disadvantaged group members; all tests are two-sided; due to a technical error, the evaluation of the majority voting system was missing for one group: Estimates are therefore based on N = 244.

Fairness ratings

The three fairness items did not exhibit satisfying internal consistency (Cronbach's α between .36 and .63), we therefore report the differences between choice systems and asymmetry conditions for each item separately. Perceptions that the process deciding over the extraction of each group member was fair were overall lower in asymmetric groups but did not differ between advantaged and disadvantaged group members (see Table S6).

Table S6. General and differential effects of choice systems and asymmetry on fairness ratings (item "In the last 6 periods, the process deciding over the extraction of each group member was fair.") with random effects for person and group (*N* = 244).

	Fixed effects						Random effects	
Predictors	*B*	*95% CI*	*SE*	*df*	*t*	*p*	*SD Person*	*SD Group*
Intercept	4.45	4.25, 4.65	0.10	492	43.52	<.001	0.00	0.59
Asymmetry	-0.58	-0.98, -0.17	0.20	60	-2.82	.007		
Advantaged	0.24	-0.11, 0.59	0.18	185	1.36	.176		
Intercept	3.50	3.21, 3.79	0.15	490	23.69	<.001	1.63	0.83
Median	1.65	1.32, 1.98	0.17	490	9.72	<.001	2.27	0.70
MVoting	1.33	0.97, 1.69	0.18	490	7.33	<.001	2.06	0.98
Intercept	3.48	3.19, 3.78	0.15	486	23.13	<.001	1.61	0.85
Median	1.63	1.29, 1.97	0.17	486	9.40	<.001	2.25	0.73
MVoting	1.27	0.92, 1.62	0.18	486	7.14	<.001	2.06	0.92
Asymmetry	-0.24	-0.84, 0.36	0.30	60	-0.80	.425		
Advantaged	0.64	0.11, 1.17	0.27	185	2.38	.018		
Median × Asym	-0.26	-0.94, 0.42	0.35	486	-0.74	.459		
Median × Adv	-0.83	-1.57, -0.10	0.37	486	-2.22	.027		
MVoting × Asym	-0.78	-1.49, -0.07	0.36	486	-2.17	.030		
MVoting × Adv	-0.36	-1.03, 0.31	0.34	486	-1.05	.293		

Note. MVoting = Majority voting, Asym = Asymmetry, Adv = Advantaged; Median and MVoting are dummy coded with individual choice as reference; Asymmetry is contrast coded to compare symmetric versus asymmetric groups; Advantaged is contrast coded to compare advantaged versus disadvantaged group members; all tests are two-sided; due to a technical error, the evaluation of the majority voting system was missing for one group: Estimates are therefore based on *N* = 244.

Both collective choice systems increased the perception that the process deciding over the extraction of each group member was fair. The analysis including the interaction of asymmetry conditions and choice systems revealed that the increase in fairness ratings in the median choice system was larger for disadvantaged group members (b = 1.96, $t(486)$ = 7.04, p

< .001) than advantaged group members (*b* = 1.13, *t*(486) = 4.04, *p* < .001). The increase in fairness ratings in the majority voting system was larger for symmetric (*b* = 1.79, *t*(486) = 8.01, *p* < .001) compared to asymmetric groups (*b* = 1.01, *t*(486) = 5.41, *p* < .001).

Ratings of the degree to which individuals felt the opportunity to influence the group results differed substantially between symmetric and asymmetric groups with higher ratings overall and a larger increase in the majority system by the former (see Table S7).

Table S7. General and differential effects of choice systems and asymmetry on fairness ratings (item "In the last six periods I had the opportunity to influence the group result.") with random effects for person and group (*N* = 244).

	Fixed effects						Random effects	
Predictors	*B*	*95% CI*	*SE*	*df*	*t*	*p*	*SD Person*	*SD Group*
Intercept	4.34	4.18, 4.51	0.08	492	51.84	<.001	0.71	0.09
Asymmetry	-0.41	-0.74, -0.07	0.17	60	-2.44	.018		
Advantaged	0.81	0.39, 1.24	0.21	185	3.80	<.001		
Intercept	3.74	3.47, 4.02	0.14	490	26.84	<.001	2.10	0.31
Median	1.11	0.77, 1.45	0.17	490	6.47	<.001	2.53	0.48
MVoting	0.80	0.41, 1.19	0.20	490	4.05	<.001	2.48	0.93
Intercept	3.73	3.46, 4.00	0.14	486	27.34	<.001	2.00	0.34
Median	1.13	0.79, 1.47	0.17	486	6.54	<.001	2.49	0.50
MVoting	0.73	0.36, 1.10	0.19	486	3.87	<.001	2.43	0.80
Asymmetry	-0.16	-0.71, 0.38	0.27	60	-0.60	.552		
Advantaged	1.68	1.02, 2.34	0.33	185	5.03	<.001		
Median × Asym	0.18	-0.50, 0.85	0.34	486	0.52	.606		
Median × Adv	-1.29	-2.11, -0.48	0.41	486	-3.12	.002		
MVoting × Asym	-0.94	-1.68, -0.20	0.38	486	-2.49	.013		
MVoting × Adv	-1.31	-2.10, -0.51	0.41	486	-3.22	.001		

Note. MVoting = Majority voting, Asym = Asymmetry, Adv = Advantaged; Median and MVoting are dummy coded with individual choice as reference; Asymmetry is contrast coded to compare symmetric versus asymmetric groups; Advantaged is contrast coded to compare advantaged versus disadvantaged group members; all tests are two-sided; due to a technical error, the evaluation of the majority voting system was missing for one group: Estimates are therefore based on *N* = 244.

Within asymmetric groups, advantaged individuals overall reported a higher perceived opportunity to influence the group result. However, both collective choice systems increased the perceived opportunity to influence the group result especially for disadvantaged group members (median choice: *b* = 1.83, *t*(486) = 6.15, *p* < .001; majority voting: *b* = 1.07, *t*(486) =

3.55, $p < .001$), and not significantly for advantaged group members (median choice: b = 0.54, $t(486) = 1.82$, $p = .070$; majority voting: b = -0.24, t(-0.76), $p = .343$).

The perception that personal motives of other group members like greed influenced participants' income did not differ between symmetric and asymmetric groups nor between advantaged and disadvantaged group members (see Table S8). Both collective choice systems reduced ratings on this item. No interactions between choice system and asymmetry condition occurred.

Table S8. General and differential effects of choice systems and asymmetry on fairness ratings (item "In the last six periods personal motives of other group members (e.g., greed) influenced my income.") with random effects for person and group (N = 244).

	Fixed effects						Random effects	
Predictors	*B*	*95% CI*	*SE*	*df*	*t*	*p*	*SD Person*	*SD Group*
Intercept	3.96	3.70, 4.22	0.13	492	29.45	<.001	0.92	0.80
Asymmetry	0.23	-0.31, 0.77	0.27	60	0.85	.398		
Advantaged	-0.41	-0.85, 0.03	0.22	185	-1.82	.070		
Intercept	4.51	4.18, 4.84	0.17	490	26.63	<.001	1.74	1.01
Median	-1.02	-1.37, -0.66	0.18	490	-5.62	<.001	2.15	0.93
MVoting	-0.69	-1.03, -0.34	0.17	490	-3.93	<.001	2.13	0.86
Intercept	4.52	4.18, 4.86	0.17	486	26.08	<.001	1.75	1.02
Median	-1.00	-1.36, -0.63	0.18	486	-5.40	<.001	2.15	0.94
MVoting	-0.67	-1.02, -0.32	0.18	486	-3.78	<.001	2.13	0.87
Asymmetry	0.10	-0.59, 0.79	0.35	60	0.28	.781		
Advantaged	-0.21	-0.78, 0.37	0.29	185	-0.72	.475		
Median × Asym	0.24	-0.49, 0.96	0.37	486	0.64	.520		
Median × Adv	-0.31	-1.01, 0.40	0.36	486	-0.85	.395		
MVoting × Asym	0.17	-0.54, 0.87	0.36	486	0.47	.642		
MVoting × Adv	-0.29	-0.99, 0.41	0.36	486	-0.82	.412		

Note. MVoting = Majority voting, Asym = Asymmetry, Adv = Advantaged; Median and MVoting are dummy coded with individual choice as reference; Asymmetry is contrast coded to compare symmetric versus asymmetric groups; Advantaged is contrast coded to compare advantaged versus disadvantaged group members; all tests are two-sided; due to a technical error, the evaluation of the majority voting system was missing for one group: Estimates are therefore based on N = 244.

Order effects of choice systems

Participants played the common resource game under each of the three choice systems. The order of choice systems was randomised. To control for order effects, we included whether participants started with individual choice or with one of the collective choice systems as covariate in the analyses. Whether participants started with individual or collective choice did not alter the effects of asymmetry, choice systems, or their interaction with reference to the average percentage taken (see Figure S2).

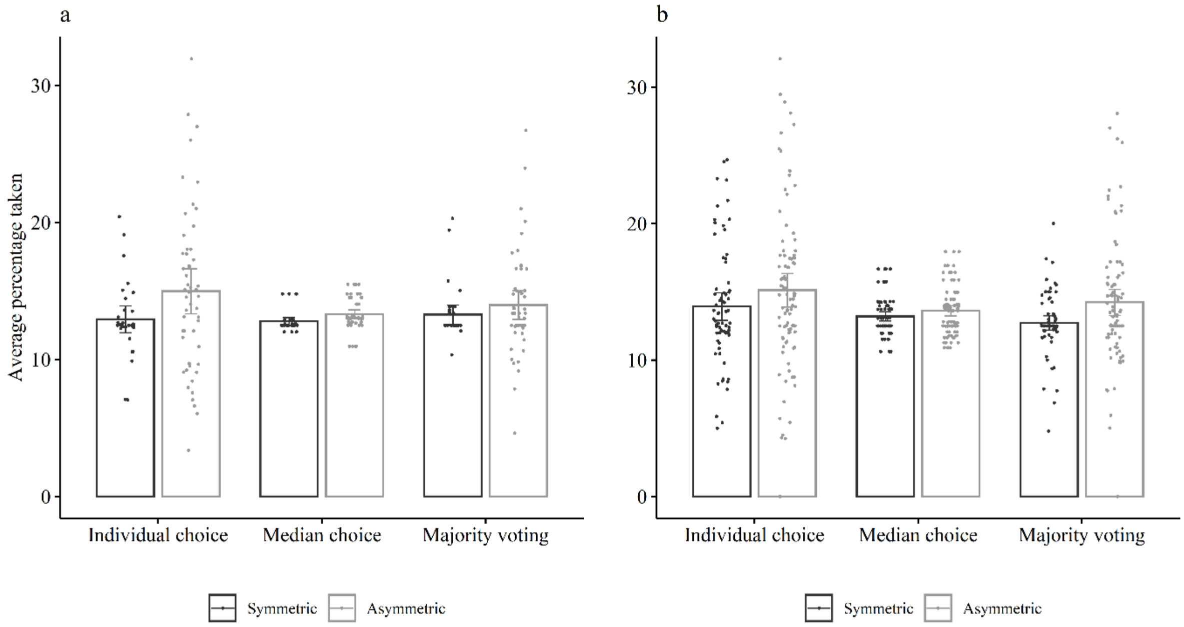

Figure S2. Average percentage taken depending on choice system and asymmetry when (a) individual choice came first, and (b) collective choice came first.

The order of choice systems did have an impact on satisfaction and fairness ratings. Asymmetric groups reported lower levels of satisfaction and fairness compared to symmetric groups, especially when they started with individual choice. In asymmetric groups, the change in satisfaction and fairness ratings between median choice and individual choice did not depend on whether groups started with individual or collective choice. The increase in satisfaction and fairness ratings for the majority voting system was larger for asymmetric

groups when they started with individual choice (*b* = 0.78, *t*(480) = 4.17, *p* < .001) compared to when they started with collective choice (*b* = 0.27, *t*(480) = 1.94, *p* = .053; see Figure S3). Symmetric groups rated median choice and majority voting more favourably than individual choice when they started with collective choice (median choice: *b* = 1.25, *t*(480) = 7.84, *p* < .001; majority voting: *b* = 1.30, *t*(480) = 8.04, *p* < .001), whereas the effect was smaller when they started with individual choice (median choice: *b* = 0.42, *t*(480) = 1.76, *p* = .078; majority voting: *b* = 0.47, *t*(480) = 1.96, *p* = .051). These results are explained by the fact that symmetric groups reported high levels of satisfaction and fairness for individual choice when it was the first choice system they experienced. It is likely that the second and third choice system were evaluated in comparison to the first choice system experienced (relative comparison), and symmetric groups corrected their positive evaluation of individual choice when comparing it to collective choice.

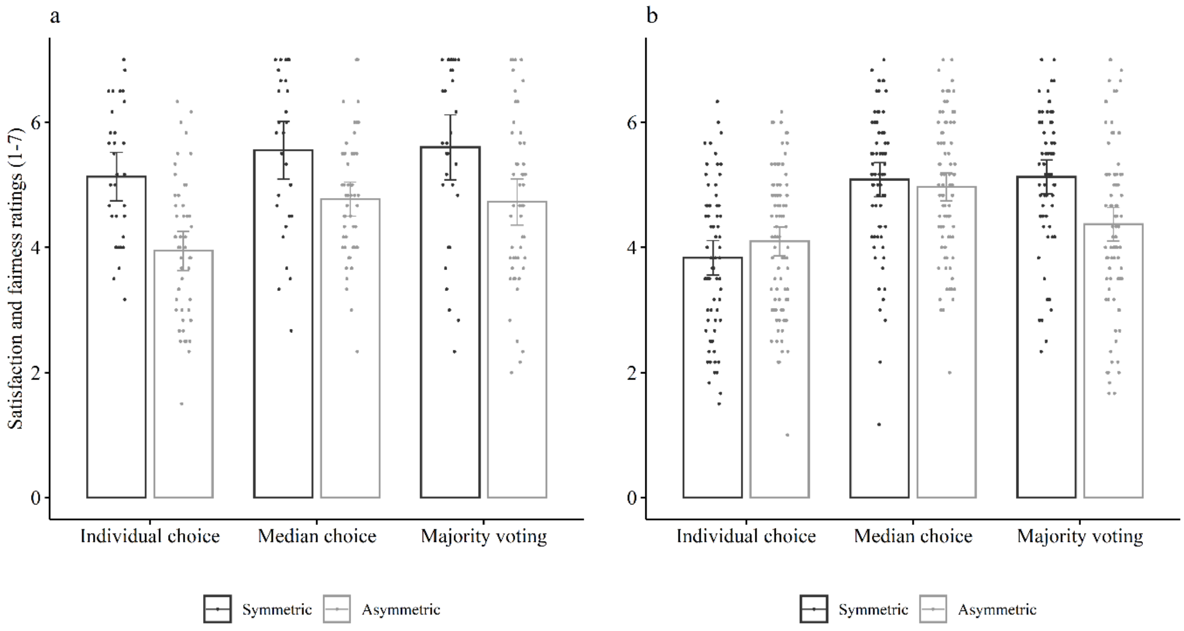

Figure S3. Satisfaction and fairness ratings depending on choice system and asymmetry when (a) individual choice came first, and (b) collective choice came first.

The order of choice systems also influenced the interplay of asymmetry and choice system in predicting profits. In symmetric groups the difference between median choice and majority voting compared to individual choice was larger when they started with one of the collective choice systems (median choice: b = 10.60, t(484) = 3.64, p < .001; majority voting: b = 11.54, t(484) = 3.96, p < .001) compared to when they started with individual choice (median choice: b = 3.84, t(484) = 0.88, p = .380; majority voting: b = 6.97, t(484) = 1.59, p = .112). In asymmetric groups, the difference between median choice and majority voting compared to individual choice was larger when they started with individual choice (median choice: b = 20.29, t(484) = 5.92, p < .001; majority voting: b = 20.98, t(484) = 6.12, p < .001), because profits were substantially lower in the individual choice when that was the first choice system (see Figure S4). When asymmetric groups started with collective choice, they earned even less in the majority voting compared to individual choice (b = -6.49, t(484) = -2.52, p = .012).

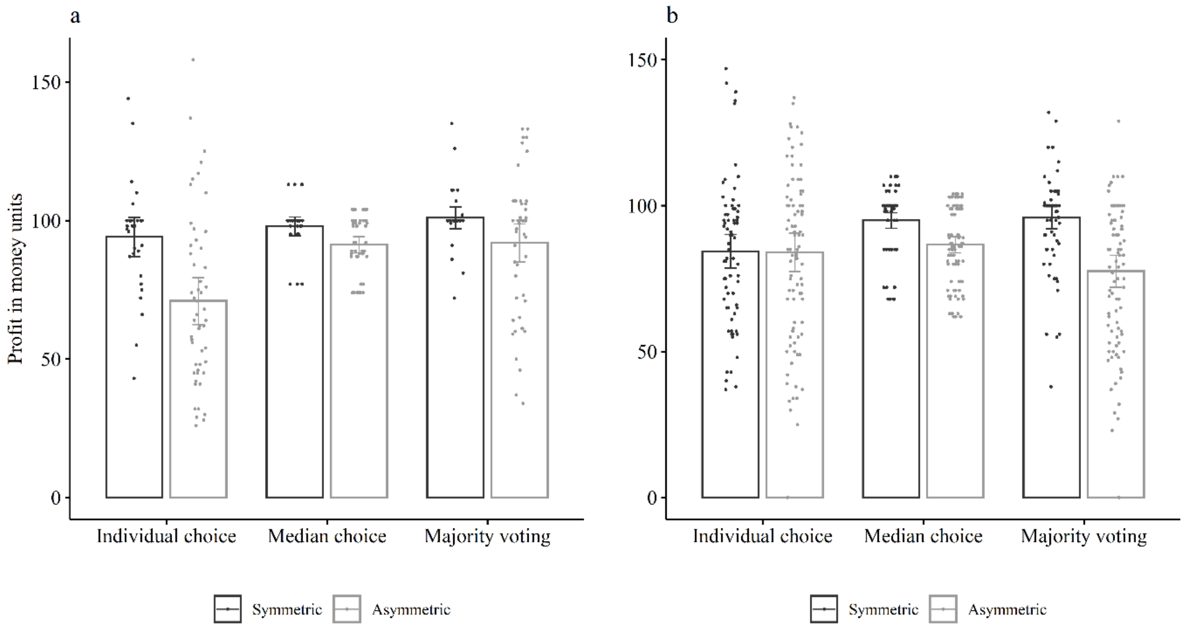

Figure S4. Profit in money units depending on choice system and asymmetry when (a) individual choice came first, and (b) collective choice came first.
